# F213. THE EFFECTS OF GROUP INTEGRATIVE ARTS THERAPY BASED ON SOCIAL SKILL TRAINING ON THE SOCIAL ADAPTIVE FUNCTION, EMPOWERMENT AND SUBJECTIVE WELL-BEING IN INPATIENTS WITH CHRONIC SCHIZOPHRENIA

**DOI:** 10.1093/schbul/sby017.744

**Published:** 2018-04-01

**Authors:** Eunsung Lim, Sang-Yeol Lee, Won-Myong Bahk, Bo-Hyun Yoon, Duk-In Jon, Moon Doo Kim, Sung-Yong Park, Min-Kyu Song

**Affiliations:** 1 Shinsegae Hospital; 2 Wonkwang University, School of Medicine; 3 Yeouido St. Mary’s Hospital, The Catholic University of Korea; 4 Naju National Hospital; 5 College of Medicine, Hallym University; 6 School of Medicine, Jeju National University; 7 Keyo Hospital; 8 Yesan Mental Health Clinic

## Abstract

**Background:**

The object of this study was to investigate the effects of group integrative arts therapy based on social skill training on communication, social adaptive function, and subjective well-being in inpatients with chronic schizophrenia.

**Methods:**

Among the 125 patients who had been hospitalized in the H mental hospital in S city after being diagnosed with schizophrenia by psychiatrists according to DSM-IV, 72 patients were selected by inclusion criteria and 48 patients were randomly assigned into an experimental group(n=16), comparative group(n=16), and control group(n=16). During this study, 4 patients from each group dropped out. The final subjects of each groups were 12 patients. The experimental group followed a 60 minutes long social skill training based on group integrative arts therapy program for twice a week and 20 times in total. The Comparative group followed a social skill training program only for 60 minutes twice a week for 20 times in total. The control group received no treatment. To assess the social adaptive function, empowerment, subjective well-being of the subjects, Communication Competence Scale(CCS), Empowerment Scale(ES) and Korean Modification of Subjective Well-Being Scale(KmSWN) were used as subjective measuring. Assertiveness Observation Evaluation Scale(AOES), Social Adaptive Functioning Scale(SAFS), and Nurses’ Observation Scale of Inpatient Evaluation-30(NOSIE-30) were also used as objective measuring that were rated by nurses or social workers at the mental hospital. One-way ANOVA and Chi-Square Test were performed to check differences among groups homogeneity. Mixed ANOVA and Sheffe test were used to find the effect of group integrative arts therapy in the differences among groups.

**Results:**

First, there was no statistically significant difference except non-verbal communication of CCS among three groups in homogeneity test of sociodemographic and clinical variables.

2nd, the group integrative arts therapy based on social skill training was found to significantly increase the communication of experimental group more than comparative group, and that of comparative group more than the control group.

3rd, the group integrative arts therapy based on social skill training was found to significantly increase the assertiveness of the experimental group and comparative group more than control groups.

4th, the group integrative arts therapy based on social skill training was found to significantly increase the social adaptive functioning of the experimental group more than comparative group, and that of the comparative group more than the groups.

5th, the group integrative arts therapy based on social skill training was found to significantly increase the NOSIE-30 of the experimental group and the comparative group more than control group. NOSIE-positive and irritability of NOSIE-30 in the comparative group was increased more than those of the experimental and the control groups.

6th, the group integrative arts therapy based on social skill training was found to significantly increase the empowerment of the experimental group more than that of the comparative and the control group.

7th, the group integrative arts therapy based on social skill training was found not to significantly increase the subjective well-being in all of the experimental, comparative and control groups.

**Discussion:**

The group integrative arts therapy based on social skill training is found to significantly enhance the social adaptive function and empowerment of inpatients with chronic schizophrenia than social skill training. These results suggest that group integrative arts therapy could be utilized as effective mental rehabilitation intervention program for inpatients with chronic schizophrenia.

